# Cardiopulmonary Exercise Testing in Congenital Heart Disease: A Never-Ending Story from Paediatrics to Adult Life

**DOI:** 10.3390/children12091175

**Published:** 2025-09-03

**Authors:** Giulia Guglielmi, Sara Moscatelli, Giorgia Rocchetti, Piergiuseppe Agostoni, Massimo Chessa, Massimo Mapelli

**Affiliations:** 1 Pediatric and Adult Congenital Heart Centre, IRCCS-Policlinico San Donato, Piazza Edmondo Malan, 2, 20097 Milan, Italy; giulia.guglielmi@grupposandonato.it (G.G.); massimo.chessa@grupposandonato.it (M.C.); 2 Centre for Inherited Cardiovascular Diseases, Great Ormond Street Hospital for Children NHS Foundation Trust, Great Ormond Street, London WC1N 3JH, UK; sara.moscatelli@gosh.nhs.uk; 3 Centre for Paediatric Inherited and Rare Cardiovascular Disease, Institute of Cardiovascular Science, University College London, Gower Street, London WC1E 6BT, UK; 4 Heart Failure Unit, Centro Cardiologico Monzino, IRCCS, 20138 Milan, Italy; giorgia.rocchetti@unimi.it (G.R.); piergiuseppe.agostoni@ccfm.it (P.A.); 5 Department of Clinical Sciences and Community Health, Cardiovascular Section, University of Milan, 20138 Milan, Italy; 6 Department of Cardiovascular Diseases, Vita Salute San Raffaele University, Via Olgettina, 58, 20132 Milan, Italy

**Keywords:** cardiopulmonary exercise test, congenital heart diseases

## Abstract

***Background:*** Cardiopulmonary exercise testing (CPET) is increasingly recognized as a key tool for evaluating patients with congenital heart disease (CHD). While traditional assessments focus on resting parameters, CPET provides dynamic, integrated insight into cardiovascular, respiratory, and muscular function during exertion. ***Objectives:*** This review explores the clinical value of CPET across the spectrum of CHD, with dedicated focus on its applications in both adult and paediatric populations. We analyse the prognostic significance of key CPET parameters—particularly peak oxygen consumption (peak VO_2_), ventilatory efficiency (VE/VCO_2_ slope), and heart rate dynamics—within distinct anatomical and physiological categories of CHD. ***Findings:*** CPET reliably detects exercise intolerance, guides intervention timing, informs exercise prescription, and stratifies risk. Peak VO_2_ and heart rate reserve are consistently associated with adverse outcomes across most CHD types. However, the prognostic utility of other variables, such as the VE/VCO_2_ slope, varies with pathophysiology—being less reliable in cyanotic lesions like Eisenmenger syndrome. In paediatric patients, CPET must be adapted to growth-related physiological variability and is increasingly used to assess quality of life, functional limitation, and response to therapy. ***Conclusions:*** CPET is a powerful, non-invasive tool that should be integrated into routine management of CHD patients across all ages. It enhances risk assessment, supports tailored care, and promotes safe physical activity, ultimately contributing to improved long-term outcomes and quality of life.

## 1. Introduction

Patients with congenital heart disease (CHD) are a constantly increasing population. Thanks to advances in care for these patients, more than 90% of children born with a congenital cardiac condition survive to adulthood.

Symptoms in this population are generally evaluated using the New York Heart Association (NYHA) functional classification. However, patients with CHD can underestimate symptoms, as they may become accustomed to a long-standing (often life-long) suboptimal functional capacity and may not perceive small variations. Therefore, the NYHA classification should be used cautiously, and objective parameters of functional capacity should be routinely measured [1].

This is where cardiopulmonary exercise testing (CPET) sets in. In fact, guidelines for CHD highlight the importance of formal exercise testing to objectively assess the quality of life and the functional capacity of this population in both adults and paediatrics [2,3]. In this setting, CPET can measure various variables, including exercise capacity through peak oxygen consumption (peak VO_2_), ventilatory efficiency through the ventilation-to-carbon dioxide output (VE/VCO_2_) slope, and chronotropic and blood pressure response (Table 1).

Moreover, it can detect symptoms on exertion, exercise-induced arrhythmias, ECG ST changes during exercise, and decreases in oxygen levels.

Baseline spirometry is also systematically performed as part of CPET, providing lung function parameters as forced expiratory volume in one second (FEV_1_), forced vital capacity (FVC), and maximum voluntary ventilation (MVV). This allows for the calculation of the breathing reserve (BR, defined as the difference between the maximal voluntary ventilation and the actual maximal ventilation at peak exercise), which is particularly relevant in CHD to distinguish cardiac from pulmonary limitations and to detect restrictive ventilatory defects, often due to prior thoracic surgery or musculoskeletal abnormalities.

Practical applications of CPET include identification of disease progression through periodic exercise capacity assessment; risk stratification to guide interventions, including transplant, in a timely fashion; and identification of the effects of previous therapeutic interventions [9]. CPET is also paramount for exercise prescription, in terms of detecting possible medical contraindications at baseline, detecting the anaerobic threshold (AT), and monitoring improvement in cardiovascular fitness through increased levels of peak VO_2_ compared to baseline [10]. This review will explore the importance and the applications of CPET in both adult and paediatric populations, giving a general overview in both populations, as well as in four different categories: biventricular circulation with a systemic morphologically left ventricle, biventricular circulation with a systemic morphologically right ventricle (RV), univentricular circulation, and Eisenmenger syndrome (ES).

## 2. Adults with Congenital Heart Disease

### 2.1. Indications and Timings

According to current AHA guidelines, CPET is recommended (Class IIa) for baseline functional assessment, risk stratification, activity counselling, and longitudinal follow-up in adults with CHD (ACHD). Institution-specific normative CPET values can be referenced, though variability exists across centres [2]. To further guide follow-up and decision-making, the AHA/ACC introduced a physiological staging system (A to D) based on functional status, haemodynamics, and clinical risk, complementing anatomical classifications [2]:

- Stage A (“at risk”)—Includes asymptomatic patients, with either repaired or unrepaired CHD, without anatomical, haemodynamic, or rhythm abnormalities who maintain normal exercise capacity and organ function.
- Stage B—Mildly symptomatic patients with mild structural or functional abnormalities, such as trivial shunts, mild valve disease or ventricular dilation, or arrhythmias that do not require treatment; objective exercise testing may reveal early limitations.
- Stage C—Patients with clinical symptoms, typically NYHA class III, due to moderate or greater valvular or ventricular dysfunction, significant shunts, or pulmonary hypertension (Arrhythmias may be present but are controlled, and organ dysfunction, if present, is generally responsive to therapy.
- Stage D—Advanced disease, characterized by severe functional limitation (NYHA IV), refractory arrhythmias, cyanosis or severe hypoxemia, Eisenmenger physiology, and end-organ dysfunction that is no longer responsive to treatment.

Based on this framework, the recommended frequency of CPET varies by lesion type and physiological stage (Table 2). For example,

- In atrial septal defects (ASDs), ventricular septal defects (VSDs), atrioventricular septal defects (AVSDs), and patent ductus arteriosus (PDA): as needed for stages A or B, every 12–24 months for stage C, and every 6–12 months for stage D;
- In mitral stenosis, subaortic and supravalvular aortic stenosis: as needed for stage A, every 2 years for stage B and C, and annually for stage D;
- In pulmonary stenosis and double-chambered RV: every 2 years for stage B and C and yearly for stage D;
- In aortic coarctation and Ebstein’s anomaly: every 3 years (stage A), every 2 years (stage B or C), or yearly (stage D);
- In tetralogy of Fallot (ToF): every 3–5 years (stage A), every 2–5 years (stage B), or every 1–2 years (stage C or D);
- In right ventricular-to-pulmonary artery (RV-PA) conduit patients: as needed (stage A/B) or every 12–24 months (stage C/D);
- In arterial switch operation: every 3–5 years (stage A/B), every 2–3 years (stage C), or every 1–2 years (stage D).

In contrast, ESC guidelines do not assign specific intervals for CPET, although they strongly recommend its use to objectively assess exercise capacity [3]. A reduction in exercise performance may indicate intervention in several ACHD conditions, such as RV-PA conduit dysfunction, RV outflow tract obstruction (RVOTO), Ebstein’s anomaly, and ToF [3]. Exercise testing is also particularly important in asymptomatic patients with moderate-to-severe valvular or supravalvular aortic stenosis, where it helps confirm true asymptomatic status and assess for exertional arrhythmias or hypotension, which are crucial factors for risk stratification and surgical timing [3].

From a practical standpoint, the preferred exercise modality in most centres is cycle ergometry, given its safety, reproducibility, and ease of monitoring (ECG and blood pressure). However, treadmill protocols are also feasible and may be particularly advantageous in selected patients, such as those with heart failure (HF) or ES, who often perceive cycling as disproportionately strenuous. Regardless of the chosen modality, consistency in testing conditions is crucial for reliable longitudinal follow-up.

### 2.2. General Considerations on CPET Parameters in Adults with Congenital Heart Disease

ACHD patients consistently exhibit a reduced peak VO_2_ compared to healthy, age-matched controls, even when classified as NYHA class I. This reduction in exercise capacity has been shown to be comparable to that observed in non-congenital patients with chronic HF, regardless of the specific type of CHD [11,12].

Due to the wide variety of congenital heart conditions and the differences in severity, even within the same diagnosis, it is unrealistic to expect that a patient with a complex cardiac condition will achieve the same peak VO_2_ as someone with a simple lesion. Also, relying on reference values from healthy individuals to interpret CPET results can be misleading in patients with CHD. To address this, Kempny et al. analysed CPET data from 4415 adults with CHD, generating percentiles for peak VO_2_ based on age, sex, and specific diagnoses. These reference curves enable clinicians to assess a patient’s exercise capacity in the context of their CHD profile, allowing for a more individualized and accurate evaluation [13]. Results demonstrated substantial variation in exercise capacity among the different ACHD groups, with ES and those with complex defects such as univentricular hearts having the lowest peak VO_2_ and the steepest VE/VCO_2_ slope, indicators of limited physical endurance. On the opposite end, patients with aortic coarctation and those with transposition of the great arteries (TGA) corrected via arterial switch showed the highest peak VO_2_ and lowest VE/VCO_2_ slope. Nevertheless, even these groups had significantly lower peak VO_2_ values compared to healthy controls.

Similarly, an important study by Inuzuka et al. showed a progressive reduction in the percentage of predicted peak VO_2_ across various CHD conditions [14]. In this cohort, 81% of patients with ES and 60% of patients with complex cyanotic heart disease did not reach 50% of the predicted peak VO_2_, in contrast with only 19% of patients with simple lesions showing such a limitation.

Another central finding in ACHD is chronotropic incompetence (CI), a condition defined as the inability of the heart rate to increase appropriately during exercise. CI is often identified when the heart rate fails to reach 85% of the age-predicted maximum or when the chronotropic index—calculated as (HRpeak − HRrest) ÷ (220 − age − HRrest)—is less than 0.8 [15]. CI is particularly prevalent in patients with complex physiologies such as systemic RV, Fontan circulation, or Ebstein’s anomaly. In Diller et al.’s cohort [16] of 64 patients with a systemic RV or univentricular circulation, CI was observed in over 70% of patients and was significantly associated with lower peak VO_2_ and shorter exercise duration. Similarly, Chen et al. [17] demonstrated that in patients with Ebstein’s anomaly and repaired tetralogy of Fallot (rToF), exercise capacity was more closely linked to chronotropic response and cardiac index than to the severity of valvular regurgitation.

Another relevant marker of functional status is the anaerobic threshold (AT), which is the point during exercise at which the body transitions from primarily using aerobic metabolism to increasingly relying on anaerobic metabolism, as the oxygen supply to the muscles is no longer sufficient to meet energy demands [4]. It can be easily calculated during a maximal, symptom-limited, ramp-protocol CPET, as previously described [18]. Kempny et al. proposed the use of AT as a tool to guide lifestyle recommendations, occupational choices, and the selection of patients for tailored rehabilitation programs, where these activities should elicit physical strain under the AT, without triggering anaerobic metabolism [13].

### 2.3. Prognostic Role of CPET

Overall, CPET plays a critical prognostic role in ACHD [7]. In a study by Dimopoulos et al., reduced peak VO_2_ (<15.5 mL/kg/min), reduced heart rate reserve (HRR—calculated as peak HR—resting HR), and elevated VE/VCO_2_ slope (>38) have all been associated with adverse cardiovascular events, including mortality and hospitalization. These prognostic markers appeared particularly significant in non-cyanotic patients; in contrast, VE/VCO_2_ slope was less predictive in cyanotic individuals, likely due to the confounding effects of increased physiological dead space [1]. These findings were subsequently confirmed in a metanalysis by Wadey et al., where peak VO_2_, HRR, and VE/VCO_2_ slope were the most predictive CPET parameters for major adverse cardiovascular events (MACE—e.g., all-cause mortality, cardiac transplantation, and hospitalization) in ACHD patients, with peak VO_2_ being inversely associated with MACE across all CHD subtypes [7].

Similarly, a study by Inuzuka et al. showed that peak VO_2_ and HRR were independent predictors of mortality in ACHD patients, but it showed that simpler parameters, such as a low resting oxygen saturation, were also linked to mortality. Notably, the combined assessment of peak VO_2_ and HRR proved to be a strong indicator of midterm mortality, likely due to their complementary prognostic roles, with peak VO_2_ primarily reflecting HF risk and HRR being more closely linked to arrhythmia-related death. This study also observed that when the respiratory exchange ratio (RER) was ≤1.0, the predictive value of both peak VO_2_ and HRR was significantly diminished. This suggests that submaximal effort during testing can limit the reliability of these parameters in assessing mortality risk in ACHD patients [14].

This prognostic role of heart rate dynamics can be extended to pregnancy in women with CHD, as demonstrated by a multicentre study by Lui et al. [19]. The study found that CI was independently associated with an increased risk of maternal cardiac events (e.g., HF and arrhythmias) and neonatal complications (e.g., prematurity and low birth weight), even when peak VO_2_ was within normal limits [19]. These findings highlight the effectiveness of heart rate response parameters in risk stratification, particularly when pregnancy requires additional haemodynamic stress on women with CHD.

### 2.4. Specific Categories

Table 3 summarizes the prognostic significance of CPET parameters across various CHD conditions.

### 2.5. Biventricular Circulation with a Systemic Morphologically Left Ventricle

This anatomical–functional category includes several congenital heart defects with preserved biventricular circulation and a morphologically left ventricle supporting systemic circulation. Within this broad group, ToF and RVOTO (including pulmonary stenosis) are among the most studied and clinically relevant conditions.

Across multiple studies, peak VO_2_ has emerged as a consistent and powerful prognostic marker in patients with rTOF. Reduced peak VO_2_ is independently associated with an increased risk of MACE, including death, hospitalization, and need for reintervention. In a large cohort of rTOF patients followed by Giardini et al., a peak VO_2_ < 36% of the predicted value and a VE/VCO_2_ slope > 39 identified individuals at significantly increased risk of cardiac-related mortality (5-year mortality of 48% and 31%, respectively), whereas no events occurred above these thresholds [20]. These findings were mirrored in Babu-Narayan et al.’s surgical series, where patients undergoing pulmonary valve replacement with a preoperative peak VO_2_ < 20 mL/kg/min had a markedly higher early mortality (5.7%), compared to 0% in those above this cutoff [22].

Longitudinal data reinforce the relevance of CPET in tracking functional decline. Kipps et al. observed that even over a relatively short follow-up period (~2.8 years), rTOF patients exhibited a progressive reduction in peak VO_2_ (from 78% to 73% of predicted), which was correlated with decreasing oxygen pulse (VO_2_/HR, which is a surrogate for stroke volume) and worsening ventilatory efficiency. Notably, these changes were not associated with static structural markers on cardiac magnetic resonance (e.g., RV volume or ejection fraction), suggesting that dynamic circulatory limitations during exertion, rather than anatomic deterioration, primarily drive functional decline in this population [23].

**Figure 1:**
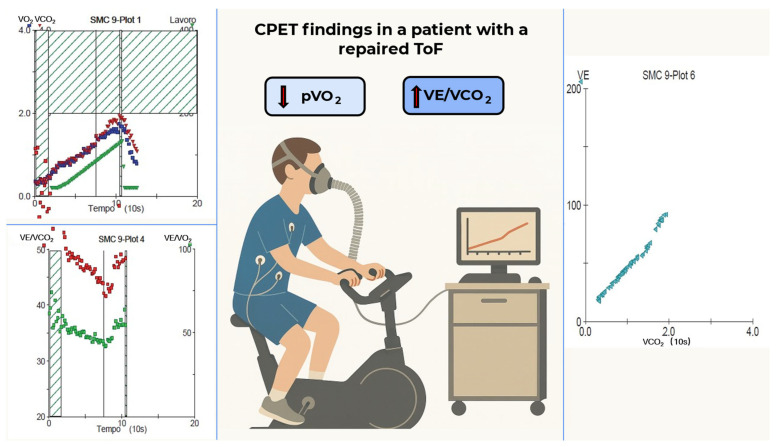
Cardiopulmonary exercise test in a patient with repaired tetralogy of Fallot. The plots show markedly reduced peak VO_2_ and a steep VE–VCO_2_ slope. Abbreviations: ToF—tetralogy of Fallot; pVO_2_—peak oxygen uptake; VE/VCO_2_—ratio of ventilation (VE) to carbon dioxide (CO_2_) production. Green symbol is work load, blue squares are the VO_2_ slope and red arrows are VCO_2_ slore.

The VE/VCO_2_ slope has also been established as a key marker of ventilatory inefficiency and adverse prognosis (Figure 1). While elevated slopes are often attributed to hyperventilation in other cardiac conditions, Mezzani et al. [24] proposed a different mechanism in repaired CHD: increased VE/VCO_2_ reflects diminished CO_2_ production, secondary to impaired perfusion rather than excess ventilation, especially in patients with RVOTO. Supporting this, they showed that ventilation levels were similar between patients with steep and normal VE/VCO_2_ slopes, but those with steeper slopes had worse gas exchange efficiency, likely due to low stroke volume or residual lesions such as pulmonary stenosis.

Taken together, these findings underscore the critical role of CPET in the long-term management of rTOF and RVOTO. Both peak VO_2_ and VE/VCO_2_ slope provide independent, prognostic information, while a declining VO_2_ trend on serial testing may indicate worsening haemodynamics or deconditioning, demanding closer surveillance or timely intervention.

### 2.6. Biventricular Circulation with a Systemic Morphologically Right Ventricle

Patients with a systemic RV in the context of biventricular circulation may have previous atrial redirection surgery (Mustard or Senning procedure) for D-transposition of the great arteries (D-TGA) or atrioventricular discordance and ventriculo-arterial discordance in the context of congenitally corrected transposition of the great arteries (ccTGA). In both cases, the result is an RV in the systemic position, which, compared to the morphologically left ventricle, is less adapted to chronic systemic pressure overload. Thus, significant decline in ventricular function usually occurs from the fourth decade onwards [25].

Also, when reported as asymptomatic or only mildly symptomatic, these patients exhibit a marked objective exercise impairment, as demonstrated by Tay et al. [26] in a study on 27 ccTGA patients, where the average peak VO_2_ was only 69% of the predicted values. These patients also had abnormal VE/VCO_2_ slopes and reduced HRR, indicating impaired functional capacity. Importantly, using tissue Doppler echocardiography, the authors found that patients with more severe exercise impairment had elevated right ventricular filling pressures, as measured by a higher E/E′ (septal and lateral annulus), a surrogate for diastolic dysfunction of the systemic RV [26].

Other than playing a key role in evaluation of exercise capacity and chronotropic response, CPET in patients with an atrial switch operation can also help identify baffle leaks through exercise-induced desaturation, which may not be apparent at rest [3].

For this category, CPET is recommended every 3–5 years for patients in stage A or B, every 1–2 years for those in stage C, and annually for those in stage D [2].

### 2.7. Univentricular Heart

In patients with a univentricular heart who have undergone a Fontan procedure, reduced exercise capacity is almost universal. This impairment stems from the peculiar physiology of the Fontan circulation, in which systemic venous return is routed directly to the pulmonary arteries without a subpulmonary ventricle. As a result, pulmonary blood flow becomes entirely passive and is highly sensitive to changes in intrathoracic pressure and central venous pressure. During exercise, these patients are unable to increase cardiac output effectively due to the absence of a pulmonary pump, impaired preload reserve, and often elevated pulmonary vascular resistance [27]. In addition, long-term consequences of Fontan physiology—such as systemic ventricular dysfunction, atrioventricular valve regurgitation, arrhythmias, and liver or lymphatic congestion—can further limit aerobic capacity. CI and autonomic dysfunction are also frequent, contributing to blunted heart rate responses during exertion [16]. Given this physiology, CPET is paramount in the longitudinal evaluation of Fontan patients.

Diller et al. [28] reported that only 2.7% of Fontan patients reached borderline peak VO_2_ (80–90% of predicted), and just 1% achieved normal values. Interestingly, no significant difference in peak VO_2_ was observed among different surgical types (atriopulmonary, atrioventricular, or extracardiac total cavopulmonary connections), perhaps indicating that functional limitation is largely inherent to Fontan physiology rather than specific anatomy. Several CPET parameters have shown prognostic significance in this group. Diller’s study found that a lower HRR, reduced peak VO_2_ (absolute and percent predicted), elevated VE/VCO_2_ slope, and lower AT were all associated with increased risk of cardiac-related hospitalization. However, for prediction of survival (freedom from death or cardiac transplantation), only HRR remained independently significant, probably due to the high prevalence of CI and arrhythmias in Fontan patients, conditions that are poorly tolerated and often drive clinical deterioration. Another study by Diller et al. showed that the prevalence of CI among various CHD conditions was highest in patients with univentricular circulation (83%) [16].

These findings were reinforced by Udholm et al. [21], who analysed seven retrospective cohort studies totalling 1664 Fontan patients (149 deaths, 35 transplants). Exercise capacity was consistently reduced (peak VO_2_ ranging from 21.2 to 27.1 mL/kg/min), but a serial decline in VO_2_ over time, rather than a single measurement, was a more reliable predictor of poor outcomes. Heart rate parameters, including peak HR and HRR, were consistently associated with both mortality and hospitalization. Also, the role of exercise oscillatory ventilation (EOV)—a pattern of cyclic fluctuations in minute ventilation during exercise—was analysed. EOV, present in 38% of patients, independently predicted both death and transplantation, even when observed during submaximal effort. The VE/VCO_2_ slope, a marker of ventilatory inefficiency, was universally elevated in Fontan patients and reliably associated with morbidity, although less consistently with mortality.

Thus, while several parameters, like peak VO_2_, VE/VCO_2_ slope, and AT, are linked to morbidity, only HRR has demonstrated consistent independent prognostic value for survival. Nonetheless, in cases where Fontan patients develop signs of HF, the 2024 International Society for Heart and Lung Transplantation guidelines consider a peak VO_2_ below 50% of the predicted value as a criterion supportive of listing for cardiac transplantation [29].

According to current recommendations, CPET for univentricular circulation should be performed every three years for stage A patients, every two years for stage B, and annually for stages C and D [2].

### 2.8. Eisenmenger Syndrome

Patients with ES are significantly impaired in their ability to exercise and demonstrate the most severe functional limitations among all CHD subgroups. Studies report that symptoms such as exertional dyspnoea and fatigue appear early in life, with more than two-thirds of patients symptomatic by age 9 and over 80% by age 28 [30].

One of the main aspects of this condition is a marked ventilatory inefficiency. The presence of a right-to-left shunt leads to the mixing of deoxygenated venous blood with systemic circulation. This results in arterial hypoxemia and leads to increased amounts of CO_2_ and hydrogen ions reaching systemic circulation without pulmonary clearance, which stimulates central and peripheral chemoreceptors, leading to an increased ventilatory drive and, thus, to hyperventilation [1]. While seemingly maladaptive, this phenomenon serves a compensatory chemical role by preserving arterial pH and pCO_2_ within near-normal ranges through enhanced CO_2_ clearance, at least during mild-to-moderate activity [4]. Other mechanisms also contribute to decreased exercise tolerance in ES patients, such as increased physiological dead space (VD/VT ratio), reduced BR, and peripheral muscle deconditioning. Relative anaemia due to iron deficiency and secondary erythrocytosis may also alter oxygen delivery and oxygen tissue utilization.

Studies by Diller et al. and Aguiar Rosa et al. showed that Eisenmenger patients exhibit profoundly reduced peak VO_2_, with mean values as low as 11.5 ± 3.6 mL/kg/min, and significantly elevated VE/VCO_2_ slopes, reflecting marked ventilatory inefficiency [1,12]. This steep slope is not only common in Eisenmenger physiology but also appears early during exercise, making it a particularly useful and reproducible parameter, even when maximal effort cannot be achieved.

This finding was further supported by a study assessing stair climbing with portable CPET in Eisenmenger patients, which showed that these patients had a more exaggerated ventilatory response, even if compared with idiopathic pulmonary arterial hypertension (IPAH) patients, and they also had a more severe dyspnoea perception for the same oxygen uptake [31]. A more recent study by Samaranayake et al. explored the prognostic value of BR during CPET in patients with ES or IPAH. A reduced BR (≤30% of predicted) was significantly associated with worse 10-year transplant-free survival in ES patients and was present in 50% of them, probably linked to structural lung restriction (e.g., elevated cardiothoracic ratio and reduced lung volumes) and increased ventilatory drive from right-to-left shunting [32].

Interestingly, while VE/VCO_2_ slope strongly predicts mortality in non-cyanotic ACHD patients, this was not observed in ES patients, despite their much higher absolute VE/VCO_2_ values (mean ~57 vs. ~33 in non-cyanotics in a study by Dimopoulos et al.) [33]. This discrepancy likely reflects a qualitative difference in pathophysiology: in ES, the elevated slope results primarily from right-to-left shunting, pulmonary hypoperfusion, and severe ventilation/perfusion mismatch rather than autonomic dysfunction or pulmonary vascular resistance alone. Therefore, the prognostic significance of CPET parameters like VE/VCO_2_ must be interpreted in the clinical context of cyanosis.

In patients with ES, CPET is indicated every 6 to 12 months for those classified as physiological stage C or D and remains crucial for guiding vasodilator therapy and assessing changes in clinical status [2].

## 3. Children with Congenital Heart Disease

### 3.1. Indications and Characteristics

Although CPET has been more extensively studied in adults with CHD, its application in children is equally relevant. Beyond measuring functional capacity, CPET also plays a key role in optimizing quality of life, particularly by supporting safe participation in daily physical activity and sports [34,35]. This functional test has also been proven to be safe in children as young as six years old, provided that protocols are adapted appropriately and that certain contraindications are excluded, such as acute myocarditis or pericarditis, severe outflow tract obstruction, and significant aortic dilation [36,37].

However, CPET in paediatric populations presents peculiar physiological, technical, and interpretive challenges that distinguish it markedly from adult testing. Unlike adults, children are in continuous stages of growth and maturation, resulting in evolving cardiopulmonary function, muscle mass, and metabolic responses that significantly affect exercise capacity and the interpretation of test outcomes [38]. Importantly, paediatric normative values are age-, sex-, and size-dependent, requiring the use of age- and sex-specific z-scores rather than fixed percent-predicted cutoffs commonly used in adults [39].

Moreover, it is important that face masks, ergometers, and sensors are size-appropriate and minimally invasive to ensure comfort and cooperation. The cycle ergometer is preferred over treadmill protocols in children due to its safety, ease of data acquisition (e.g., ECG and BP), and reduced mechanical demands [34]. As an alternative to the Bruce protocol, the Godfrey protocol with ramp modifications is frequently employed for children, offering gradual workload increases suitable for smaller and deconditioned individuals [40]. Innovative approaches such as age-appropriate outdoor running protocols with mobile metabolic equipment have been shown to be feasible, even in very young children with CHD, starting from age 4, helping to address the diagnostic gap in this age group and enabling functional assessment in a more natural, engaging environment than traditional laboratory-based testing [41].

Finally, paediatric CPET interpretation must account for differences in symptom perception and communication. Children may struggle to articulate symptoms like dyspnoea or chest discomfort and might terminate tests for subjective reasons such as unfamiliarity or fear, rather than physiological limitation. This necessitates greater reliance on objective parameters and clinician observation to assess exertional capacity and underlying pathophysiology.

### 3.2. General Considerations on CPET Parameters in Children with Congenital Heart Disease

Amedro et al. conducted one of the largest multicentre prospective studies evaluating cardiopulmonary fitness in children with CHD, comparing 766 CHD patients to 357 healthy controls [42]. Overall, peak VO_2_ was significantly reduced in CHD patients compared to healthy peers (33.4 ± 8.7 vs. 44.6 ± 8.5 mL/kg/min, *p* < 0.001). The most severe impairments were observed in patients with cyanotic CHD, with mean peak VO_2_ as low as 21.6 ± 5.9 mL/kg/min. Children with rToF had moderately impaired performance (mean VO_2_ 32.0 ± 7.2 mL/kg/min), while those with simple lesions such as ASD or isolated pulmonary stenosis approached near-normal values (~40–42 mL/kg/min).

Similarly, ventilatory efficiency was worse in CHD patients, with the VE/VCO_2_ slope significantly elevated (median 30.2 [IQR 27.1–34.5] vs. 27.1 [24.9–29.6] in controls, *p* < 0.001), especially in cyanotic and single-ventricle groups (>34). Oxygen pulse was also reduced across all CHD groups, and chronotropic incompetence was prevalent, particularly in Fontan and Eisenmenger patients [42].

CPET variables can also predict health-related quality of life (HR-QoL), as demonstrated by Amedro et al. in a large prospective multicentre cohort of 202 children aged 8–18 years with CHD [43]. Patients were stratified according to CHD severity into four categories, from mild (class I) to complex, uncorrectable, or palliated conditions (class IV), with 63% of participants falling into the most severe classes (III–IV). Notably, peak VO_2_, % predicted peak VO_2_, and % predicted VO_2_ at AT showed significant correlations with both self-reported and parent-reported physical well-being scores. In contrast, the VE/VCO_2_ slope did not correlate with any HR-QoL dimensions. Importantly, parents’ proxy reports of physical well-being were more strongly associated with objective CPET metrics than children’s self-reports, especially in younger participants [43].

Finally, a sensitive index of aerobic exercise function in children with CHD was explored by Reybrouck et al [44]. In a cross-sectional study, 29 patients having undergone an atrial switch operation for TGA and 30 patients with rTOF were compared with 24 age-matched healthy controls. The authors found that the slope of VO_2_ versus exercise intensity (expressed in mL O_2_/min^2^/kg) was significantly lower in CHD patients (1.50 ± 0.64 in TGA and 1.68 ± 0.75 in TOF, compared to 2.42 ± 0.68 in healthy controls (*p* < 0.001)). Patients also exhibited a steeper VE/VCO_2_, reflecting ventilatory inefficiency. Importantly, subnormal VO_2_ versus exercise intensity slopes (<1.8) were associated with significant residual haemodynamic lesions such as moderate-to-severe pulmonary regurgitation in TOF and subvalvular stenosis [44].

Thus, routine use of CPET in the paediatric CHD population should be encouraged to objectively monitor exercise capacity, as well as to avoid unnecessary restrictions and promote safe, active lifestyles [37].

### 3.3. Future Perspective and Unmet Needs

The functional assessment of patients with CHD remains an evolving field, with several important gaps that warrant further research. CHD is, by definition, a dynamic and lifelong condition; its clinical course and functional capacity can change substantially over time due to disease progression, residual lesions, or secondary complications. As in HF, prognostic evaluation should not rely solely on a single “snapshot” provided by one CPET [45]. Instead, longitudinal assessment of CPET-derived variables, capturing both improvement and deterioration, may help delineate distinct trajectories that may accurately predict patient outcomes and guide management strategies [7].

However, access to high-quality CPET in CHD care is hindered by limited availability in many centres; a lack of standardized, patient-tailored protocols; insufficient numbers of clinicians and physiologists trained in both CPET and CHD; and accreditation processes that are less rigorous than those for echocardiography. Additionally, protocols should be adapted to the patient’s underlying anatomy and functional capacity, and CHD teams should receive targeted education on the indications, interpretation, and integration of CPET results into comprehensive physiological assessment [46].

Although cycle ergometer CPET remains the gold standard for functional testing, it may not fully replicate the physical demands experienced by CHD patients in daily life, which often involve different muscle groups and movement patterns. This mismatch can obscure functional limitations that only manifest in real-world contexts. In HF populations, wearable metabolic measurement systems and activity monitors have already been extensively studied as a means to capture habitual functional performance and complement or even replace laboratory CPET in risk stratification [47,48,49]. In CHD, however, this concept was only preliminarily explored by Larsson et al., who compared self-reported physical activity using the International Physical Activity Questionnaire (IPAQ) with an accelerometer–heart rate system, showing that questionnaires tend to overestimate habitual activity relative to objective monitoring [50]. This further highlights the need for more patient-centred, real-world assessment approaches in CHD.

In HF, prognostic scores, such as the MECKI score [51], integrate CPET variables with echocardiographic and laboratory parameters, providing a multidimensional and systematic evaluation of disease severity. Given the complexity of CHD, a similar integrated approach seems equally essential, combining functional, imaging, and biochemical data to improve prognostic accuracy. Opotowsky et al. emphasised that existing CHD risk models are largely event- and short-term-focused, offering limited individual predictive power [52]. Thus, comprehensive, adaptable, lifespan-oriented tools that integrate anatomical, functional, genetic, and social determinants are necessary to enable truly personalised risk stratification.

Finally, given the systemic nature of CHD, functional impairment often extends beyond the heart, involving pulmonary, peripheral muscular, and other organ systems. Integrating conventional CPET data with advanced “complex CPET” techniques, such as non-invasive cardiac output and stroke volume measurement during exercise (e.g., Physioflow^®^), or peripheral oxygen extraction assessment via near-infrared spectroscopy (NIRS) could help quantify the relative contributions of central and peripheral limitations. This deeper physiological characterisation, as already explored in selected cardiac populations, holds particular promise in CHD for identifying the predominant limiting factors and tailoring interventions, whether cardiac-targeted therapies or peripheral conditioning and rehabilitation [53,54].

## 4. Conclusions

CPET provides essential, objective insight into the functional status of patients with CHD. It complements resting evaluations by revealing limitations during exertion, guiding clinical decisions across all age groups and CHD types. Parameters like peak VO_2_ and HRR are the most consistent predictors of prognosis, while others—like VE/VCO_2_ slope—require context-specific interpretation. In both adults and children, CPET supports individualized care and risk stratification and promotes safe physical activity. Its routine use should be encouraged to improve long-term outcomes and quality of life in this growing population.

## Figures and Tables

**Table 1:** Description of the principal CPET variables and reference values based on established guidelines and major studies. Abbreviations: VO_2_—oxygen uptake; VE/VCO_2_—ratio of ventilation (VE) to carbon dioxide (CO_2_) production; AT—anaerobic threshold; HRR—heart rate reserve.

CPET Parameter	Description	Normal Values
**Peak VO_2_**	The maximal O_2_ uptake during exercise, reflecting global aerobic capacity	**>85%** of predicted (age-, sex-, and body size-adjusted); typically **30–40 mL/kg/min** in healthy young adults [4,5,6]
**VE/VCO_2_**	Ratio of ventilation to CO_2_ output, indicating ventilatory efficiency	**<30** in healthy adults. Values **>34–36** indicate ventilatory inefficiency; **>38** strongly predicts poor outcomes in HF and CHD [4,5,7]
**Anaerobic Threshold (AT)**	Exercise intensity or the VO_2_ level at which metabolism shifts from aerobic to anaerobic pathways	Normally reached at **40–60% of predicted VO_2_** max, corresponding to 11–14 mL/kg/min in healthy individuals [4,6,8]
**Heart Rate Reserve (HRR)**	Difference between peak and resting heart rate, reflecting chronotropic response	**>72–80 bpm** or achieving **≥80–85%** of the age-predicted maximum HR is considered normal. Chronotropic index **≥0.8** [4,8]

**Table 2:** Recommended frequency of CPET in ACHD. Abbreviations: ASD—atrial septal defect; VSD—ventricular septal defect; AVSD—atrioventricular septal defect; PDA—patent ductus arteriosus; DRCV—double-chambered right ventricle; CoA—aortic coartation; ToF—tetralogy of Fallot; RV-PA—right ventricle-to-pulmonary artery conduit.

CHD Type	Stage A	Stage B	Stage C	Stage D
**ASD—VSD—AVSD—PDA**	As needed	As needed	12–24 months	6–12 months
**Mitral stenosis** **Subaortic stenosis** **Supravalvular aortic stenosis**	As needed	2 years	2 years	12 months
**Pulmonary stenosis** **DCRV**	As needed	2 years	2 years	12 months
**CoA** **Ebstein’s anomaly**	3 years	2 years	2 years	12 months
**ToF**	3–5 years	2–5 years	1–2 years	1–2 years
**RV-PA conduit**	As needed	As needed	12–24 months	12–24 months
**Arterial switch operation**	3–5 years	3–5 years	2–3 years	1–2 years
**Systemic RV**	3 years	3 years	2 years	12 months
**Univentricular circulation**	3 years	2 years	12 months	12 months
**Eisenmenger syndrome**	-	-	6–12 months	6–12 months

**Table 3:** Summary of the prognostic significance of the main CPET parameters across different congenital heart disease phenotypes. Abbreviations: VO_2_—oxygen uptake; LV—left ventricle; RV—right ventricle; VE/VCO_2_—ratio of ventilation (VE) to carbon dioxide (CO_2_); AT—anaerobic threshold; HRR—heart rate reserve production; ccTGA—Congenitally Corrected Transposition of the Great Arteries.

CHD Type	Peak VO_2_	VE/VCO_2_ Slope	Anaerobic Threshold (AT)	Heart Rate Reserve (HRR)
**Biventricular circulation with systemic LV** **(e.g., rTOF)**	~71% predicted [13]; prognostic cut-off ≤36% predicted [20]	~37 [13]; prognostic cut-off >39 [20]	Often reduced	Frequently reduced. Low HRR is an independent predictor of adverse outcomes
**Biventricular circulation with systemic RV**	~63–67% predicted [13]	~ 35 [13]	Reduced	Frequently reduced; chronotropic incompetence is very common
**Fontan circulation**	~59% predicted [13]; Absolute, 21–27 mL/kg/min; serial decline more predictive than single value [21]	~34 [13]; elevated but less consistent for mortality	Reduced; inconsistent prognostic value across studies	Frequently reduced; strong predictor of mortality, transplant risk, and hospitalization
**Eisenmenger syndrome**	~42% predicted [13]; absolute, 11–17 mL/kg/min [1]	~57 [1]; highest VE/VCO_2_ among CHD groups	Markedly reduced; early onset during exercise	Frequently reduced; chronotropic incompetence is very common

## Abbreviations

ACHD	Adult Congenital Heart Disease
ASD	Atrial Septal Defect
AT	Anaerobic Threshold
AVSD	Atrioventricular Septal Defect
BR	Breathing Reserve
ccTGA	Congenitally Corrected Transposition of the Great Arteries
CHD	Congenital Heart Disease
CI	Chronotropic Incompetence
CPET	Cardiopulmonary Exercise Testing
D-TGA	Dextro-Transposition of the Great Arteries
EOV	Exercise Oscillatory Ventilation
ES	Eisenmenger Syndrome
FEV_1_	Forced Expiratory Volume in one second
FVC	Forced Vital Capacity
HF	Heart Failure
HR	Heart Rate
HRQoL	Health-Related Quality of Life
HRR	Heart Rate Reserve
IPAH	Idiopathic Pulmonary Arterial Hypertension
MACE	Major Adverse Cardiovascular Events
MVV	Maximum Voluntary Ventilation
NYHA	New York Heart Association
PDA	Patent Ductus Arteriosus
PH	Pulmonary Hypertension
RER	Respiratory Exchange Ratio
RV	Right Ventricle
RV-PA	Right Ventricle to Pulmonary Artery
RVOTO	Right Ventricular Outflow Tract Obstruction
SpO_2_	Peripheral Oxygen Saturation
TGA	Transposition of the Great Arteries
ToF	Tetralogy of Fallot
rToF	repaired ToF—Tetralogy of Fallot
VCO_2_	Carbon Dioxide Output
VD/VT	Dead Space to Tidal Volume Ratio
VE	Minute Ventilation
VE/VCO_2_ slope	Ventilation to Carbon Dioxide Output Slope
VE/VO_2_	Ventilation to Oxygen Uptake Slope
VO_2_	Oxygen Consumption
VO_2_ peak/Peak VO_2_	Peak Oxygen Consumption
VSD	Ventricular Septal Defect
